# Antioxidant allocation modulates sperm quality across changing social environments

**DOI:** 10.1371/journal.pone.0176385

**Published:** 2017-05-04

**Authors:** Alfonso Rojas Mora, Magali Meniri, Ophélie Gning, Gaëtan Glauser, Armelle Vallat, Fabrice Helfenstein

**Affiliations:** 1 Laboratory of Evolutionary Ecophysiology, Institute of Biology, Faculty of Sciences, University of Neuchâtel, Neuchâtel, Switzerland; 2 Neuchatel Platform of Analytical Chemistry, Institute of Chemistry, Faculty of Sciences, University of Neuchâtel, Neuchâtel, Switzerland; Centre National de la Recherche Scientifique, FRANCE

## Abstract

In promiscuous species, male reproductive success depends on their ability to mate with fertile females and on the fertilizing ability of their sperm. In such species, theory predicts that, owing to a trade-off between pre- and post-copulatory reproductive traits, males with lesser access to females should increase resource investment into those sperm traits that enhance fertilization success–usually referred to as ejaculate quality. This prediction has been validated in several taxa, yet studies on the physiological mechanisms modulating ejaculate quality are lacking. Sperm cells are highly vulnerable to oxidative stress, which impairs male fertility. Therefore, males that better protect their sperm from oxidative stress are expected to achieve higher ejaculate quality. Based on theoretical expectations, and since social dominance is a major determinant of mating opportunity, we predicted that subordinate males should invest more into the antioxidant protection of their sperm in order to achieve higher ejaculate quality. We maintained 60 male and 60 female wild-caught house sparrows *Passer domesticus* in outdoor aviaries, where we experimentally manipulated male social status to test our predictions. We measured cellular oxidative stress and enzymatic antioxidant activity in blood and sperm both before and after manipulating social ranks. Before manipulating the social status, we found that ejaculate viability correlated with oxidative stress level in sperm, with dominant males producing more oxidized and less viable ejaculates. Further, males at the lower end of the hierarchy produced ejaculates of similar quality to those of dominant males, suggesting that restricted access to resources might limit male reproductive strategies. After experimentally manipulating the social status, males matched their ejaculate quality to their new rank, while increases in antioxidant investment into ejaculates paralleled increases in ejaculate viability. Oxidative stress has been proposed as a general constraint to the evolution of life histories. Our results highlight oxidative stress and strategic antioxidant allocation as important proximate physiological mechanisms underlying male reproductive strategies.

## Introduction

Sexual selection arises both before copulation because males differ in their ability to access fertile females, and after copulation inside the female reproductive tract when the sperm of two or more males compete to fertilize the ova, a circumstance referred to as sperm competition [1]. Under sperm competition, the fertilizing ability of an ejaculate determines the reproductive success of a male [2, 3]. Thus, selection acts upon those ejaculate traits (e.g. sperm velocity, proportion of motile sperm, ATP production, etc.; generally referred to as ejaculate quality) that maximize fertilization success (reviewed in [4, 5, 6]). Several theoretical models have explored how much males are selected to invest into post-copulatory traits, i.e. ejaculate quality and competitiveness, given variation in their ability to mate with fertile females (reviewed in [3, 7]). These models predict a negative correlation between ejaculate quality and social dominance. The predictions of those models have been tested in several taxa [8–13], although only discrete social roles have been tested so far (e.g. favoured vs. disfavoured). Recent models predict that continuously increasing costs to obtain a mate should select for progressive increases of resource investment in the ejaculate [14, 15], resulting in a continuous trade-off between somatic vs. germline functions. Some evidence exists in support to those models, such as Engqvist’s [16] finding of a negative genetic co-variation between attractiveness and mating investment in the scorpionfly (*Panorpa cognata*). Remarkably, although several studies have investigated the potential outcomes of a soma vs. germline allocation trade-off, the actual resources to be strategically allocated to the germline are yet to be identified.

Oxidative stress (OS) is the unbalance between pro-oxidants and antioxidants [17], and has been hypothesized to be a main cost of reproduction [18, 19]. Sperm cells are especially prone to OS [20], and OS has been shown to be deleterious to sperm [21–25]. Various studies have shown that ejaculate quality correlates with the level of oxidative stress in the ejaculate [23, 26, 27], while antioxidant supplementation seems to improve ejaculate quality [28–31]. Consequently, OS has been identified as an important cause of male sub- and infertility [20, 24, 32] and may thus act as a selective pressure in shaping various sperm traits. For example, a comparative analysis has shown that across mammals the proportion of polyunsaturated fatty acids in the sperm membrane, which renders sperm membranes prone to OS [33], negatively correlates with the species level of sperm competition [34]. Here, we propose that antioxidants are the resource being traded off between somatic and germline functions, and consequently, strategic antioxidant allocation in the ejaculate should modulate ejaculate quality.

In species where dominance determines access to fertile females we predict that ejaculate quality should positively correlate with antioxidant allocation in the ejaculate, while OS should be negatively correlated with ejaculate quality. We further predict that males occupying lower social ranks should invest more antioxidant resources to the production of higher quality ejaculates. In contrast, more dominant males should invest less antioxidant resources, and thus produce more oxidatively stressed and lower quality ejaculates (the oxidation-based soma vs. germline allocation trade-off hypothesis). However, it is worth noting that in many instances males at the lower end of the hierarchy pay higher costs of subordination compared to the costs paid by dominant males to maintain their status (reviewed in [35]), and males at the bottom-end of the hierarchy might be unable to invest as much antioxidant resources to produce higher quality ejaculates. Under such scenario, the most subordinate males may be predicted to produce low quality ejaculates.

We tested the above predictions in wild House Sparrows (*Passer domesticus*), a socially monogamous passerine bird that forms social hierarchies, where more dominant males have greater access to fertile females [36]. Further, rates of extra-pair paternity in house sparrows range between 12–15% [37–40], and more dominant males better protect their females from sexual harassment by other males [36]. We tested our predictions by maintaining 15 groups of four males and four females for four weeks in outdoor aviaries. In order to test whether patterns of antioxidant allocation to the ejaculate were causally related to a males’ social dominance, we then experimentally changed the social rank of males by shuffling them across groups and aviaries to test whether changes in antioxidant allocation would match adjustments of ejaculate quality.

## Materials and methods

### Experiment setup

We trapped a total of 60 male and 60 female house sparrows using mist-nets in western Switzerland during the first two weeks of April 2014. Upon trapping, we measured body mass and tarsus length, and visually assessed the badge size of the males. Birds were then transferred into 15 mixed outdoor aviaries at the Ethological Station Hasli, University of Bern, Switzerland. Birds were distributed among the aviaries according to their body weight and an initial score of badge size, so that aviaries contained on average birds of the same body weight and males with various badge sizes. After four weeks, all the females were transferred into a separate aviary, and we took a sperm sample from each male. A second sperm sample was taken the day after, and a third sperm sample after 4 days of being sexually rested. This procedure ensured that any differences in sperm characteristics would be intrinsic differences in quality rather than differences due to depletion [8] or fresh sperm effects [41–43] (but see [44]), and only data collected after all males were manually depleted was used. For logistic reasons, males were divided in three sampling batches consisting of 5 aviaries, and all three batches were processed 5 days apart.

To test the causality of a relationship between social status, sperm quality and antioxidant allocation, females were reintroduced to the aviaries and males were shuffled across aviaries according to their initial social rank. We maximized the number of positions that males could have gained or lost in the hierarchy. Males were given three weeks to settle down the new hierarchical positions for 18 days before being re-sampled following the same procedure as before. The exact duration of spermatogenesis is unknown in house sparrows. However, spermatogenesis has been estimated to last between 11 and 15 days in non-passerine birds such as domestic fowls, Japanese quails and Barbary drakes [45], and a study by Bat & Maiti [46] on yellow-throated sparrows *Petronia xanthocollis* suggests that it may be shorter in passerine birds. We thus assumed that an 18-day period would cover at least one spermatogenesis cycle.

### Social dominance

To assess the hierarchy and rank the males in each aviary, we recorded a total of 13 hours of observations before the manipulation and 10 hours after the manipulation in each aviary. We removed the feeders for 1.5 hours, and then recorded all the antagonistic interactions at the feeders for one hour after reintroducing the feeders into the aviaries. Such feeder made any spilt seeds inaccessible, and thus birds had to compete for the two feeding sites at the seed dispenser. Within each aviary, we used interaction dyads (82 dyads per aviary on average, range 31–235 before the manipulation; 100 dyads per aviary on average, range 39–233 after the manipulation), to compute each male's David's score as a proxy for their social rank [47]. This resulted in a linear dominance hierarchy within each group (aviary) in which the most dominant males were referred to as dominant, and males lower in the social ladder were referred to as subordinate-1, subordinate-2 and subordinate-3, subordinate-3 males being at the bottom-end of the hierarchy.

### Ejaculate quality

We gently massaged the males' cloaca to obtain ejaculates [48] that were collected in glass capillaries. We took a photo of the capillary over a millimetre paper to assess ejaculate volume. 0.25 μL of ejaculate were diluted in 40 μL of preheated (40°C) Dulbecco Modified Eagle Medium (DMEM). 3 μL of this sperm-DMEM mix were loaded into a 20-μm deep swimming chamber (Leja®, The Netherlands), and sperm mobility was video recorded using a Toshiba CMOS HD camera (Toshiba Co., Japan) mounted on an Olympus BX43 microscope (Olympus Co., Japan) with a 10x objective under negative phase contrast (position Ph3 of the annular phase ring). We maintained the temperature of the sperm-DMEM mix at 40°C using a heating glass plate (MATS-U55S, Olympus Co., Japan) fitted to the microscope stage. A subsample of the ejaculate was diluted in PBS and stored at -80°C for subsequent laboratory analyses (as described in [49]). The sperm-DMEM mix was further used to assess ejaculate size (number of sperm cells) and density (in millions of sperm cells/mL) using a Neubauer counting chamber.

From the videos, we used a Computer Assisted Sperm Analyser plug-in [50] for ImageJ [51] to assess ejaculate viability (% of motile sperm) and the mean values for VCL (curvilinear velocity, total distance travelled, μm/s), VAP (average path velocity, smoothed path using roaming average, μm/s), VSL (straight line velocity, distance from origin to end point, μm/s), linearity (LIN: VSL ⁄ VAP, path curvature), wobble (WOB: VAP⁄ VCL, side to side movement of the sperm head, also described as the oscillation of the actual trajectory about its average path), BCF (beat cross frequency, the frequency at which VCL crosses VAP, Hz), and progression (PROG: average distance from origin on the average path during all frames analysed). Sperm having a VSL<5 μm/s, a VCL<15 μm/s, or a VAP<10 μm/s were assumed to be either moved by drift or immotile. These estimates were based on 71 ***±*** 37 sperm tracks (mean ***±*** SD) per ejaculate.

Ejaculate viability (% of motile sperm) and sperm swimming velocity are determinant components of male fertility and sperm competitive ability [52, 53]. Therefore, was assessed sperm performance as (1) ejaculate viability (% of motile sperm) and (2) PC1 scores from a principal component analysis with varimax rotation of the other seven variables plus the number of sperm cells detected by the CASA software. This axis captured 61.4% of the variance and was positively correlated with VSL, VCL, VAP, WOB and PROG (0.73 < r <0.98, P < 0.0001), negatively correlated with BCF (r = -0.79, P < 0.0001) and uncorrelated with LIN and the number of tracks (-0.1 < r < 0.05, P > 0.30). Hence, this first principal component axis, hereafter referred to as “sperm swimming ability”, described sperm swimming fast and efficiently (fewer overall movements to achieve greater progression).

We modelled the rate at which initial speed decreases through time (from now on referred to as “sperm swimming endurance”) as well as the rate at which initial proportion of swimming sperm decreases through time (from now on referred to as “ejaculate longevity”) using mixed linear models with time as a fixed effect and individual identity as a random factor. For further details, refer to the supplementary material.

### Oxidative stress and antioxidant defences

Antioxidant defences and an individual’s redox balance are characterised by a multidimensional system integrating several lines of antioxidant defences and oxidative damage to various biomolecules [54, 55]. Thus, and as advocated by [56], we chose to describe individual redox status through (1) a specific marker of oxidative damage to the lipids, (2) a marker of cellular oxidative stress, i.e. the proportion of oxidised over reduced glutathione (GSSG/GSH ratio), as well as GSSG and GSH by themselves to better understand the causes of variation in the proportion, and (3) the activity of the antioxidant enzyme SOD, i.e. an endogenous antioxidant that catalyses the dismutation of superoxide anions into molecular oxygen or hydrogen peroxide. We assessed these markers in two somatic tissues, i.e. the plasma and the red blood cells, and in a tissue of the germline function, i.e. the ejaculate.

We assessed the amount of lipid peroxidation by determining the levels of malondialdehyde (MDA) in plasma, red blood cells (RBCs), and sperm. MDA levels were determined by derivatisation with thiobarbituric acid and further separation by ultra-high pressure liquid chromatography (UHPLC) with fluorescent detection (as described in [49]). Standards were run in duplicates, and were highly repeatable (linear mixed model, intra-class correlation coefficient, r = 0.99 [57]). The repeatability of plasma samples was estimated in the same species, but from another dataset, and was equally high (r = 0.90).

We also measured superoxide dismutase (SOD) activity per ml of tissue in sperm and RBCs applying minor modifications to a commercial kit (Cayman Chemical, USA). All the samples were assayed in duplicates yielding low intra-plates CVs (10.7% for sperm and 9.9% for erythrocytes). Additionally, the inter-plate repeatability was assessed using 23 erythrocyte and 50 sperm samples, and was found to be high for both tissues: r = 0.89 and r = 0.85, respectively.

Finally, we determined the levels of glutathione, an intracellular antioxidant in both its reduced (GSH) and oxidized (GSSG) forms both in sperm and RBCs using UHPLC-MS/MS (following [49]). Repeatability was assessed using standards from different HPLC runs, and was high for both GSH and GSSG, r = 0.976 and r = 0.945 respectively.

### Statistical analyses

For the above-mentioned reasons, the data used for all the analyses corresponds to the last sampling, where males were sexually rested and previously sperm depleted. We used linear mixed models to test our hypotheses. We first predicted that ejaculate quality and antioxidant allocation would vary according to social ranks. In a first set of statistical models we investigated ejaculate quality and OS markers as a function of the initial social rank, including body weight and tarsus length as covariates. The dependent variables describing sperm quality were ejaculate viability, sperm swimming ability, ejaculate longevity, sperm swimming endurance, ejaculate size, and ejaculate density. The dependent variables describing antioxidant allocation and oxidative stress were the proportion of oxidized glutathione [GSSG/(GSSG+GSH), used as a measure of the oxidative stress endured by the cells in sperm and RBCs], SOD activity in sperm and RBCs, the proportion of SOD activity in the sperm relative to the total SOD activity in sperm and RBCs [SOD_sperm_ /(SOD_sperm + RBC_)], used as a measure of relative SOD investment into spermatic vs. somatic functions, MDA levels in the sperm and plasma, and the proportion of MDA in the sperm relative to the total level of MDA in sperm and plasma [MDA_sperm_/(MDA_sperm + plasma_)], used as a measure of relative oxidative stress in the sperm vs. soma.

Second, we predicted that males would adjust sperm quality and antioxidant allocation in accordance to an experimental change in their social status. To test such hypothesis, we ran a set of models that used the same set of dependent variables as mentioned above, but measured after the experimental manipulation of the social ranks. The tested factors were the initial social rank, the final social rank and their interaction, as well as final body mass and tarsus length as covariates.

Proportions were logit-transformed, while other dependent variables were log-transformed to match normality. All the models included the aviary and the date at which the males were sampled as random factors, using the restricted maximum likelihood method for parameter estimation, and Kenward-Roger approximation for the computation of fixed effects degrees of freedom. Tests of fixed effects were based on SAS Type-II tests of hypothesis. To avoid inflating the type I error [58, 59], we did not apply model selection, and therefore always report results for full models. Post-hoc tests were conducted using Tukey-Kramer adjustment. The analyses were performed using SAS v. 9.3.

### Ethical note

Animal manipulations were performed as quickly as possible to minimise stress. We recorded any injuries or anomalous behaviours that could indicate excessive pain or stress and would require euthanizing the animal according to our guideline. The veterinary office of the Canton Bern, Switzerland, after supervision and approval by the Cantonal ethical committee, authorized the experimental setup and detention conditions under licenses n° BE41/12 and WTH/g-525/14.

## Results

### Ejaculate quality

#### Before experimentally manipulating the hierarchy

**Fig 1:**
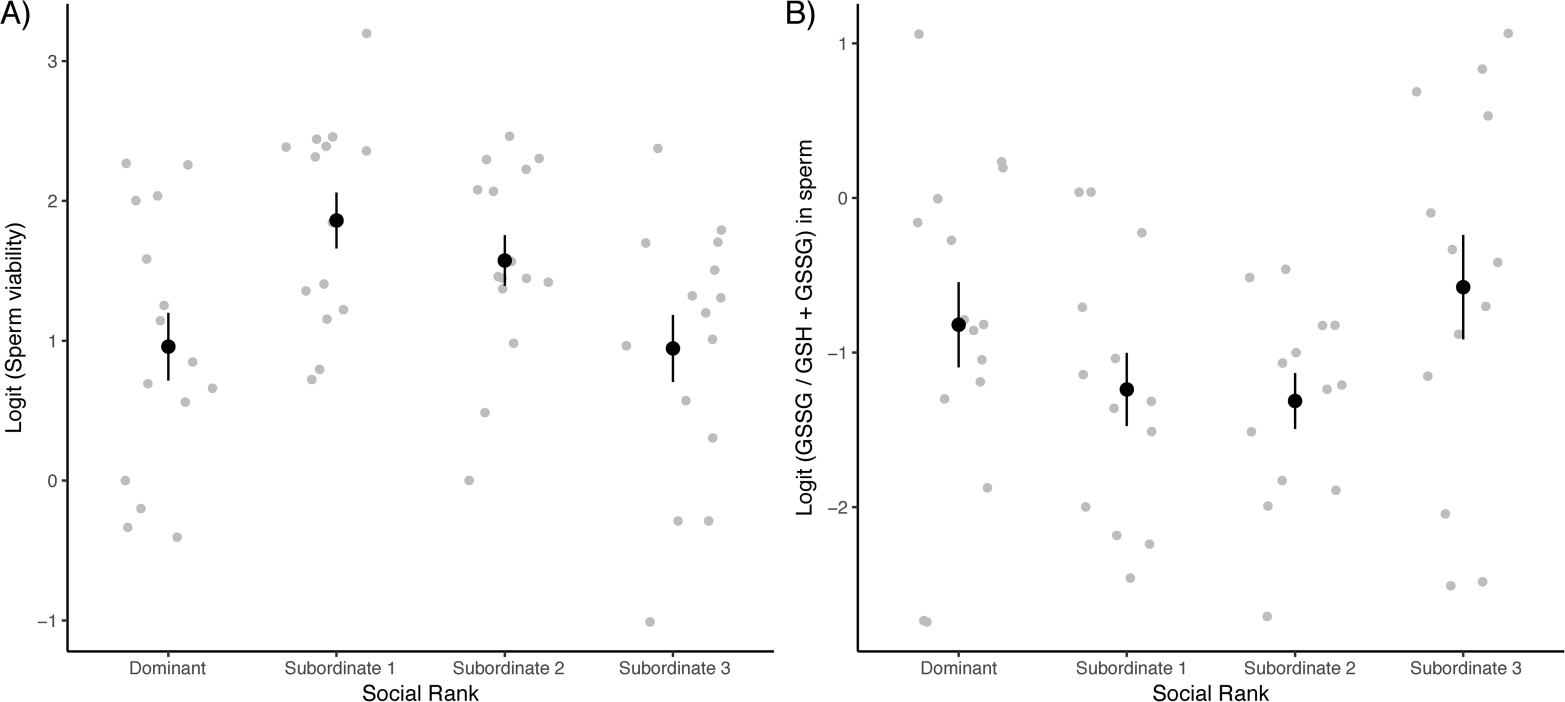
(A) Mean proportion of motile sperm ± SE (■) and (B) proportion of oxidized over total glutathione (GSSG/GSH+GSSG) in sperm ± SE (□) of males with different social status. Ejaculates with higher motility have lower proportions of GSSG, while the greater the proportion of GSSG (e.g. the more oxidatively stressed the sperm cells) the less motile the ejaculate is. Proportions are logit-transformed.

**Fig 2:**
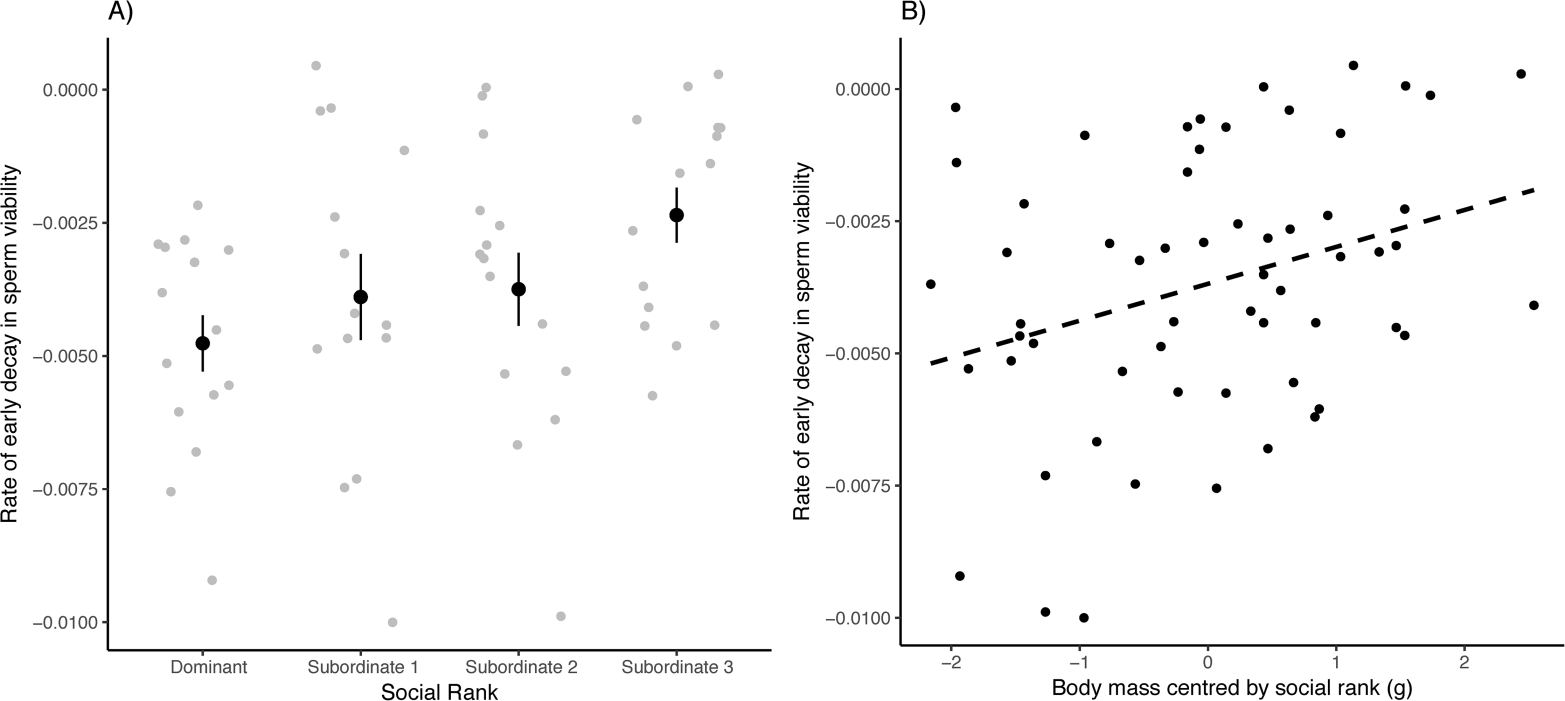
Ejaculate longevity expressed as the rate of decay in motile sperm (A) differs among males with different social status (raw data, mean ± SE), (B) while heavier males have longer living sperm (F_1,49.4_ = 7.30, p = 0.009, slope = 0.074 ± 0.026). The dotted line represents a linear regression.

Before experimentally manipulating the hierarchy, body mass and tarsus length were uncorrelated (F_1,54.4_ = 2.70, p = 0.11). The social rank was also not associated with body mass (F_3,56_ = 1.63, p = 0.19), but was associated with tarsus length (F_3,42_ = 3.57, p = 0.02). In particular, post-hoc Tukey-Kramer pairwise comparisons show that dominant males had longer tarsi than subordinate-2 males (p = 0.01). We could not collect any ejaculate from one male having a very small cloacal protuberance, thus we obtained a total of 59 ejaculates. We found that ejaculates produced by males with different social ranks differed in their initial viability (% of motile sperm) (Fig 1A; Table 1), with subordinate males 1 and 2 producing more viable ejaculates than dominant and subordinate 3 males (pairwise t-tests; p-values in bold retain significance after Tukey-Kramer correction: D vs. S1: t_40.5_ = -3.09, **p = 0.004**; D vs. S2: t_42_ = -2.30, p = 0.03; D vs. S3: t_40.7_ = -0.25, p = 0.80; S1 vs. S2: t_40.7_ = 0.70, p = 0.49; S1 vs. S3: t_40.3_ = 2.94, **p = 0.006**; S2 vs. S3: t_39.8_ = 2.23, p = 0.032). Additionally, we found that ejaculate longevity also covaried with social rank (Fig 2A; Table 1), with subordinate 3 males having ejaculates retaining viability for a longer time immediately after collection compared to that of dominant males (Tukey-Kramer adjusted post-hoc tests; D vs. S1: p = 0.41; D vs. S2: p = 0.26; D vs. S3: p = 0.026; S1 vs. S2: p = 0.98; S1 vs. S3: p = 0.50; S2 vs. S3: p = 0.75). Finally, heavier males produced longer-living ejaculates (Fig 2B; Table 1). We found no differences in sperm swimming ability or sperm swimming endurance across males with different social ranks (Table 1). Lastly, we found no effect of social rank, body mass or tarsus length on ejaculate density (social rank: F_3,51_ = 1.13, p = 0.35; body mass: F_3,51_ = 0.62, p = 0.43; tarsus length: F_3,51_ = 2.19, p = 0.15) or ejaculate size (social rank: F_3,51_ = 1.42, p = 0.25; body mass: F_3,51_ = 2.51, p = 0.12; tarsus length: F_3,51_ = 1.35, p = 0.25).

**Table 1:** LMMs investigating how social status affects ejaculate viability (% motile sperm), sperm swimming ability, ejaculate longevity and sperm swimming endurance.

*a) Ejaculate viability*				
** Random effects**	***Estimates ± SE***		**Z**	**P**
Aviary	*0*.*006* ***±*** *0*.*079*		0.07	0.47
Sampling batch	*0*.*084* ***±*** *0*.*119*		0.71	0.24
** Fixed effects**		**F**	**df**	**P**
*Intercept*	*-2*.*452* ***±*** *3*.*977*			
Body mass	*-0*.*002* ***±*** *0*.*095*	0.01	1,49.1	0.98
Tarsus length	*0*.*182* ***±*** *0*.*177*	1.06	1,50.9	0.31
**Social status**^a^		**4.68**	**3,40.6**	**0.007**
*Dominant*	*-0*.*077* ***±*** *0*.*305*			
*Subordinate-1*	*0*.*885* ***±*** *0*.*301*			
*Subordinate-2*	*0*.*672* ***±*** *0*.*301*			
*b) Sperm swimming ability*				
**Random effects**	***Estimates ± SE***		**Z**	**P**
Aviary	*0* ***±*** *0*		0	1
Sampling batch	*0*.*194* ***±*** *0*.*241*		0.80	0.21
**Fixed effects**		**F**	**df**	**P**
*Intercept*	*-5*.*189* ***±*** *4*.*659*			
Body mass	*0*.*141****±*** *0*.*112*	1.59	1,51.5	0.21
Tarsus length	*0*.*071****±*** *0*.*205*	0.12	1,51.1	0.73
Social status^a^		1.12	3,51.1	0.35
*Dominant*	*-0*.*466* ***±*** *0*.*367*			
*Subordinate-1*	*-0*.*205* ***±*** *0*.*363*			
*Subordinate-2*	*0*.*214****±*** *0*.*363*			
*c) Ejaculate longevity*				
**Random effects**	***Estimates ± SE***		**Z**	**P**
Aviary	*0* ***±*** *0*		0	1
Sampling batch	*0* ***±*** *0*		0	1
**Fixed effects**		**F**	**df**	**P**
*Intercept*	*-0*.*024* ***±*** *0*.*011*			
**Body mass**	*0*.*0007* ***±*** *0*.*0003*	**7.30**	**1,49.4**	**0.009**
Tarsus length	*0*.*0001* ***±*** *0*.*0005*	0.04	1,51	0.85
**Social status^a^**		**2.98**	**3,41**	**0.042**
*Dominant*	*-0*.*0026* ***±*** *0*.*0009*			
*Subordinate-1*	*-0*.*0012* ***±*** *0*.*0009*			
* Subordinate-2*	*-0*.*0009* ***±*** *0*.*0009*			
*d) Sperm swimming endurance*				
**Random effects**	***Estimates ± SE***		**Z**	**P**
Aviary	*0* ***±*** *0*		0	1
Sampling batch	*0* ***±*** *0*		0	1
**Fixed effects**		**F**	**df**	**P**
*Intercept*	*0*.*438* ***±*** *0*.*707*			
Body mass	*-0*.*009* ***±*** *0*.*017*	0.25	1,53	0.62
Tarsus length	*-0*.*029* ***±*** *0*.*031*	0.85	1,53	0.36
Social status^a^		0.53	3,53	0.67
*Dominant*	*-0*.*013* ***±*** *0*.*057*			
*Subordinate-1*	*0*.*038* ***±*** *0*.*056*			
*Subordinate-2*	*-0*.*028* ***±*** *0*.*056*			

^a^Relative to subordinate-3 males. Values in bold indicate significance at α = 0.05; tests of random effects are based on Wald-Z.

#### After experimentally manipulating the hierarchy

After having experimentally changed the social rank of males, body mass and tarsus length were uncorrelated (F_1,57_ = 1.94, p = 0.06). The social rank was neither associated with body mass (F_3,55_ = 0.94, p = 0.43) nor with tarsus length (F_3,55_ = 1.51, p = 0.22). After having experimentally changed the social rank of males, we could only obtain ejaculates from 59 males.

**Fig 3:**
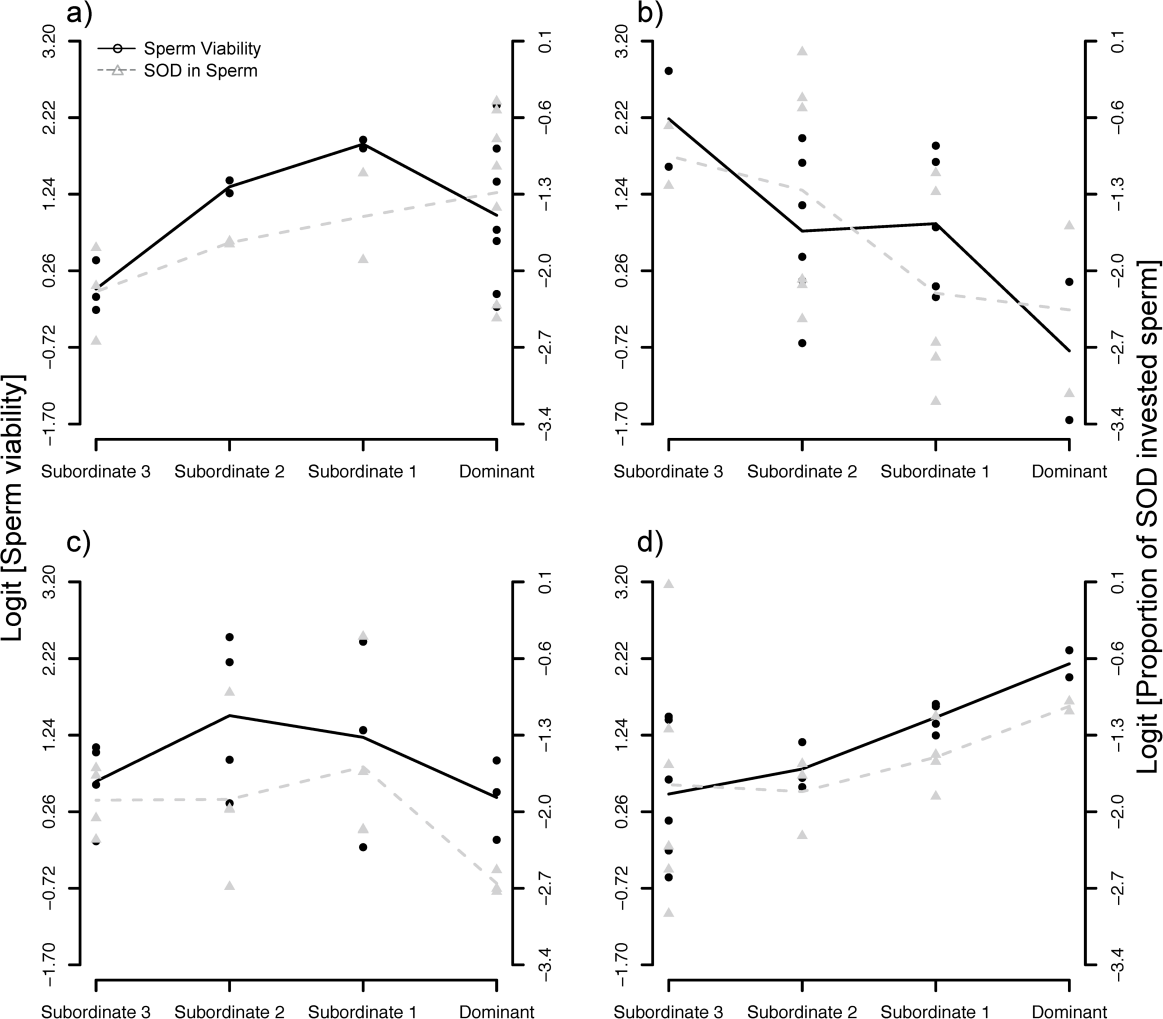
Proportion of motile sperm (black solid line and dots) and proportion of SOD activity in sperm relative to the total SOD_sperm + RBCs_ (grey dotted line and triangles) after shuffling males across aviaries. This relative sperm SOD activity is used as a proxy for the relative investment of antioxidant resources in soma vs. germline functions. Initial male social status: (a) dominant, (b) subordinate-1, (c) subordinate-2, and (d) subordinate-3 males. The lines connect the mean of each group, and are only illustrative. Proportions are logit-transformed.

We found that ejaculate viability differed among males in their new social ranks, and this depended on their initial social rank (Fig 3A–3D; Table 2). More specifically, we found bell-shaped patterns for initially dominant and initially subordinate-2 males (Fig 3A and 3C), while initially subordinate-1 males increased their ejaculate viability when going *down* the hierarchy, thus fully matching Tazzyman *et al*.’s [15] and Parker *et al*.'s (14) predictions (Fig 3B). Finally, males at the bottom of the hierarchy—subordinate-3 males—increased their sperm quality when going *up* the hierarchy (Fig 3D). Initial swimming speed, ejaculate longevity and sperm swimming endurance were neither affected by the final rank nor the interaction between the initial and final rank (Table 2). Finally, we could not test whether males adjusted ejaculate volume and density due to a technical problem.

**Table 2:** LMMs investigating how experimentally changing the social status affects ejaculate viability (% motile sperm), sperm swimming ability, ejaculate longevity and sperm endurance.

*a) Ejaculate viability*				
**Random effects**	***Estimates ± SE***		**Z**	**P**
Aviary	*0*		0	1
Sampling batch	*0*		0	1
**Fixed effects**		**F**	**df**	**P**
*Intercept*	*1*.*57* ***±*** *4*.*49*			
Body mass	*-0*.*05* ***±*** *0*.*10*	0.24	1, 41	0.63
Tarsus length	*0*.*01* ***±*** *0*.*20*	< 0.01	1, 41	0.95
Initial status^a^		0.66	3, 41	0.58
*Dominant*	*-0*.*44* ***±*** *0*.*61*			
*Subordinate-1*	*1*.*66* ***±*** *0*.*68*			
*Subordinate-2*	*0*.*10* ***±*** *0*.*55*			
Final status^a^		1.87	3, 41	0.15
*Dominant*	*1*.*73* ***±*** *0*.*73*			
*Subordinate-1*	*0*.*97* ***±*** *0*.*53*			
*Subordinate-2*	*0*.*26* ***±*** *0*.*60*			
**Initial status x Final status**^b^		**2.82**	**9, 41**	**0.011**
*Dominant* x dominant	*-0*.*79* ***±*** *0*.*93*			
*Dominant x subordinate-1*	*0*.*79* ***±*** *0*.*94*			
*Dominant x subordinate-2*	*1*.*02* ***±*** *0*.*96*			
*Subordinate-1 x dominant*	*-4*.*67* ***±*** *1*.*11*			
*Subordinate-1 x subordinate-1*	*-2*.*27* ***±*** *0*.*89*			
*Subordinate-1 x subordinate-2*	*-1*.*65* ***±*** *0*.*92*			
*Subordinate-2 x dominant*	*-1*.*92* ***±*** *0*.*94*			
*Subordinate-2 x subordinate-1*	*-0*.*43* ***±*** *0*.*79*			
*Subordinate-2 x subordinate-2*	*0*.*65* ***±*** *0*.*87*			
*b) Sperm swimming ability*				
**Random effects**	***Estimates ± SE***		**Z**	**P**
Aviary	*0*.*21* ***±*** *0*.*25*		0.86	0.19
Sampling batch	*0*.*04* ***±*** *0*.*13*		0.31	0.38
**Fixed effects**		**F**	**df**	**P**
*Intercept*	*3*.*72* ***±*** *5*.*28*			
Body mass	*0*.*16* ***±*** *0*.*11*	1.91	1, 33.2	0.18
Tarsus length	*-0*.*04* ***±*** *0*.*22*	0.03	1, 30.9	0.87
Initial status^a^		1.05	3, 36.3	0.38
*Dominant*	*0*.*67* ***±*** *0*.*74*			
*Subordinate-1*	*0*.*14* ***±*** *0*.*84*			
*Subordinate-2*	*0*.*23* ***±*** *0*.*67*			
Final status^a^		0.86	3, 24.1	0.48
*Dominant*	*-0*.*79* ***±*** *0*.*82*			
*Subordinate-1*	*0*.*53* ***±*** *0*.*61*			
*Subordinate-2*	*-0*.*37* ***±*** *0*.*66*			
Initial status x Final status^b^		0.93	9, 31.7	0.51
*Dominant* x dominant	*-0*.*25* ***±*** *1*.*06*			
*Dominant x subordinate-1*	*-0*.*94* ***±*** *1*.*08*			
*Dominant x subordinate-2*	*-0*.*91* ***±*** *1*.*15*			
*Subordinate-1 x dominant*	*2*.*41* ***±*** *1*.*31*			
*Subordinate-1 x subordinate-1*	*-0*.*06* ***±*** *1*.*02*			
*Subordinate-1 x subordinate-2*	*0*.*36* ***±*** *1*.*11*			
*Subordinate-2 x dominant*	*0*.*77* ***±*** *1*.*12*			
*Subordinate-2 x subordinate-1*	*-0*.*50* ***±*** *0*.*96*			
*Subordinate-2 x subordinate-2*	*0*.*65* ***±*** *0*.*99*			
*c) Ejaculate longevity*				
**Random effects**	***Estimates ± SE***		**Z**	**P**
Aviary	*0*		0	1
Sampling date	*0*		0	1
**Fixed effects**		**F**	**df**	**P**
*Intercept*	*-0*.*0195* ***±*** *0*.*0097*			
Body mass	*0*.*0004* ***±*** *0*.*0002*	3.70	1, 33.9	0.06
Tarsus length	*0*.*0003* ***±*** *0*.*0004*	0.53	1, 3.1	0.47
Initial status^a^		1.16	3, 36.6	0.34
*Dominant*	*0*.*0003* ***±*** *0*.*0014*			
*Subordinate-1*	*0*.*0010* ***±*** *0*.*0015*			
*Subordinate-2*	*0*.*0007* ***±*** *0*.*0012*			
Final status^a^		1.49	3, 26.4	0.24
*Dominant*	*-0*.*0012* ***±*** *0*.*0015*			
*Subordinate-1*	*-0*.*0003* ***±*** *0*.*0011*			
*Subordinate-2*	*-0*.*0008* ***±*** *0*.*0012*			
Initial status x Final status^b^		1.20	9, 32.7	0.33
*Dominant* x dominant	*0*.*0006* ***±*** *0*.*0019*			
*Dominant x subordinate-1*	*0*.*0004* ***±*** *0*.*0020*			
*Dominant x subordinate-2*	*-0*.*0016* ***±*** *0*.*0021*			
*Subordinate-1 x dominant*	*0*.*0039****±*** *0*.*0024*			
*Subordinate-1 x subordinate-1*	*-0*.*0012* ***±*** *0*.*0019*			
*Subordinate-1 x subordinate-2*	*-0*.*0002* ***±*** *0*.*0020*			
*Subordinate-2 x dominant*	*0*.*0013* ***±*** *0*.*0020*			
*Subordinate-2 x subordinate-1*	*-0*.*0014* ***±*** *0*.*0018*			
*Subordinate-2 x subordinate-2*	*0*.*0016* ***±*** *0*.*0018*			
*d) Sperm swimming endurance*				
**Random effects**	***Estimates ± SE***		**Z**	**P**
Aviary	*0*.*0011* ***±*** *0*.*0023*		0.50	0.31
Sampling date	*0*.*0039* ***±*** *0*.*0049*		0.80	0.21
**Fixed effects**		**F**	**df**	**P**
*Intercept*	*-0*.*164* ***±*** *0*.*636*			
Body mass	*-0*.*010* ***±*** *0*.*014*	0.50	1, 37.4	0.49
Tarsus length	*0*.*006* ***±*** *0*.*027*	0.04	1, 35.2	0.84
Initial status^a^		2.35	3, 36.2	0.09
*Dominant*	*0*.*107* ***±*** *0*.*088*			
*Subordinate-1*	*-0*.*033* ***±*** *0*.*101*			
*Subordinate-2*	*0*.*075* ***±*** *0*.*080*			
Final status^a^		2.68	3, 29	0.07
*Dominant*	*0*.*028* ***±*** *0*.*101*			
*Subordinate-1*	*0*.*069* ***±*** *0*.*074*			
*Subordinate-2*	*0*.*077* ***±*** *0*.*082*			
Initial status x Final status^b^		1.45	9, 35.7	0.20
*Dominant* x dominant	*-0*.*093* ***±*** *0*.*130*			
*Dominant x subordinate-1*	*-0*.*151* ***±*** *0*.*133*			
*Dominant x subordinate-2*	*-0*.*173* ***±*** *0*.*139*			
*Subordinate-1 x dominant*	*-0*.*277* ***±*** *0*.*161*			
*Subordinate-1 x subordinate-1*	*-0*.*118* ***±*** *0*.*125*			
*Subordinate-1 x subordinate-2*	*0*.*011* ***±*** *0*.*134*			
*Subordinate-2 x dominant*	*-0*.*188* ***±*** *0*.*136*			
*Subordinate-2 x subordinate-1*	*-0*.*221* ***±*** *0*.*114*			
*Subordinate-2 x subordinate-2*	*-0*.*177* ***±*** *0*.*122*			

^a^Relative to subordinate-3 males.

^b^Relative to subordinate-3 x subordinate-3 males. Values in bold indicate significance at α = 0.05; tests of random effects are based on Wald-Z.

### Oxidative stress and antioxidant allocation

#### Before experimentally manipulating the hierarchy

**Fig 4:**
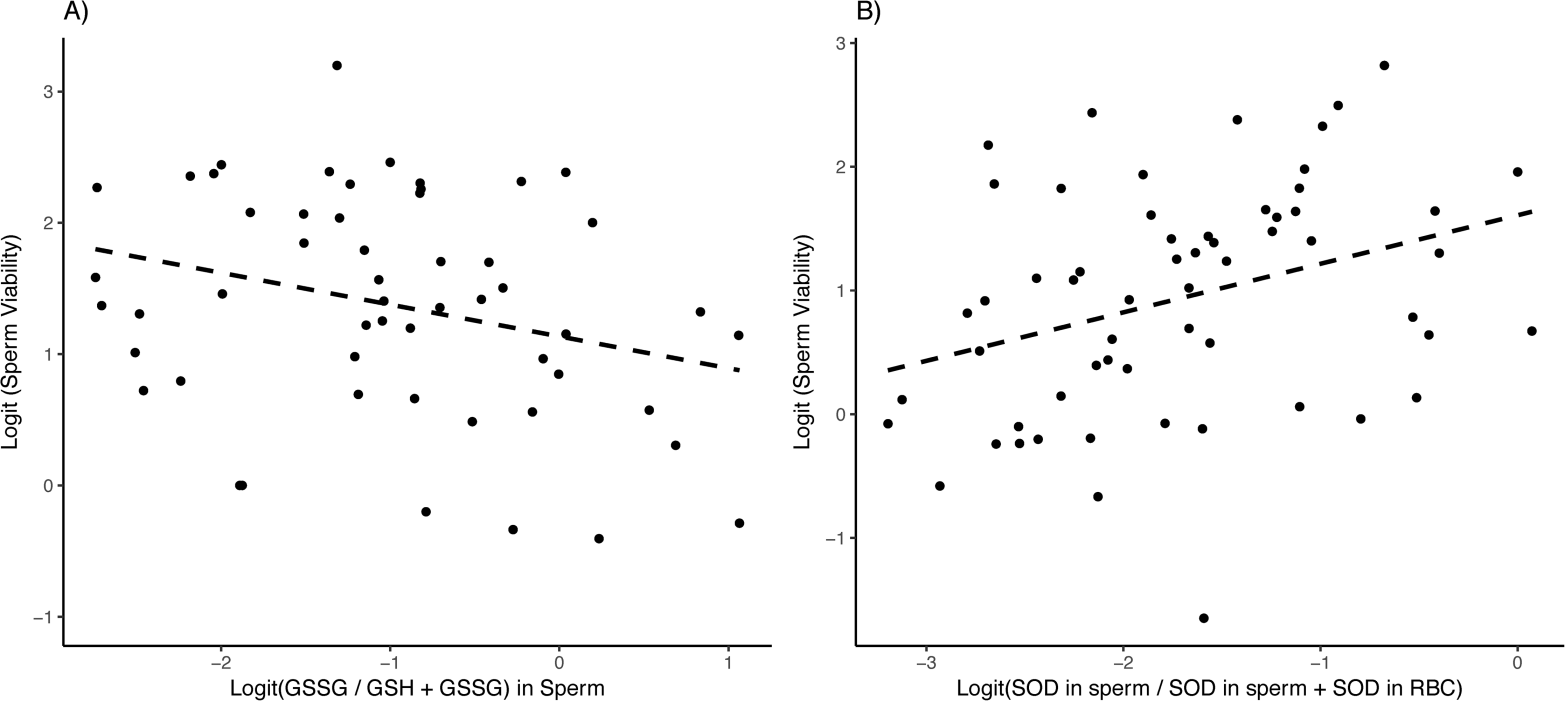
Relationship between the proportion of motile sperm with (A) the proportion of oxidized over total glutathione (GSSG/GSH+GSSG) (F_1,50.8_ = 4.67, p = 0.036, slope = -0.25 ± 0.11) before manipulating the social status, and (B) the proportion of SOD in sperm relative to total SOD activity (SOD_sperm_/SOD_sperm + RBCs_) (F_1,56.9_ = 7.78, p = 0.007; slope = 0.43 ± 0.15) after manipulating the social status. The dotted lines represent linear regressions.

We found that the proportion of oxidized over total concentration of glutathione in the ejaculate varied according to the social status (Fig 1; social status: F_3,48_ = 2.96, p = 0.04; body mass: F_48.5_ < 0.01, p = 0.98; tarsus length: F_48.1_ = 0.63, p = 0.43), with dominant and subordinate 3 males having more oxidatively stressed sperm cells (pairwise t-test; none of the p-values retain significance after Tukey-Kramer correction: D vs. S1: t_48.1_ = 1.83, p = 0.07; D vs. S2: t_48_ = 1.89, p = 0.065; D vs. S3: t_48.2_ = -0.45, p = 0.65; S1 vs. S2: t_48_ = 0.20, p = 0.84; S1 vs. S3: t48 = -2.29, p = 0.027; S2 vs. S3: t_48_ = -2.43, p = 0.019). Moreover, we found that the higher the proportion of oxidized over total concentration of glutathione in the sperm (the more oxidized the sperm cells) the lower the proportion of motile sperm in the ejaculate (Fig 4A; F_1,50.8_ = 4.67, p = 0.036, slope = -0.25 ± 0.11). The ratio between oxidized over total glutathione in the RBCs did not differ across social ranks (social status: F_3,54_ = 0.74, p = 0.53; body mass: F_54_ 0.76, p = 0.39; tarsus length: F_54_ = 0.14, p = 0.71).

Absolute concentrations of SOD activity and GSH in either sperm or RBC did not differ across social ranks (all F ≤ 3.25, p ≥ 0.078). Nor did the proportion of SOD in sperm relative to the SOD_sperm + RBCs_ or the proportion of reduced glutathione GSH in sperm relative to GSH_sperm + RBCs_ (all F ≤ 1.60, p ≥ 0.21). The levels of damage to lipids did not differ across males with different social ranks in any of the sampled tissues (all factors and covariates: F ≤ 0.54, p ≥ 0.46). The proportion of MDA in sperm relative to plasma or RBC did also not differ across social ranks (all factors and covariates F ≤ 1.23, p ≥ 0.27), which indicates that variation in sperm quality is achieved through differential allocation of antioxidant resources, but at constant levels of oxidative damage both in the soma and the germline. Samples sizes and degrees of freedom vary across measures due to technical problems with the different assays.

### After experimentally manipulating the hierarchy

Our manipulation of the social status did not affect the levels of SOD activity in the soma (Table 3). Interestingly, males with a longer tarsus showed higher SOD activity in their red blood cells (Table 3). We found that both the levels of SOD activity in sperm (Table 3) and the proportion of SOD activity in sperm relative to the SOD activity in the soma depended on the final social rank in relation to the initial rank (Fig 3A–3D; Table 3). Moreover, both sperm SOD activity (F_1,56.9_ = 11.34, p = 0.0014; slope = 0.55 ± 0.16) and the proportion of SOD activity in sperm (Fig 4B; F_1,56.9_ = 7.78, p = 0.007; slope = 0.43 ± 0.15) positively correlated with the proportion of motile sperm. No other marker of oxidative stress or antioxidant allocation (absolute or relative levels of MDA or GSH in sperm or blood) responded to our manipulation (all F ≤ 3.62, p ≥ 0.06).

**Table 3:** LMMs investigating how experimentally changing the social status affects the levels of SOD activity in RBCs, SOD activity in sperm, and the proportion of SOD in sperm relative to total SOD activity (sperm + RBCs).

*a) SOD activity in RBCs*				
**Random effects**	***Estimates ± SE***		**Z**	**P**
Aviary	*0*.*03* ***±*** *0*.*02*		1.22	0.11
Sampling batch	*0*.*01* ***±*** *0*.*01*		0.08	0.47
**Fixed effects**		**F**	**df**	**P**
*Intercept*	*3*.*85* ***±*** *1*.*72*			
Body mass	*0*.*04* ***±*** *0*.*04*	1.24	1, 35.3	0.27
**Tarsus length**	***0*.*15 ± 0*.*07***	**4.42**	**1, 33.8**	**0.04**
Initial status^a^		1.42	3, 37.3	0.26
*Dominant*	*0*.*20* ***±*** *0*.*24*			
*Subordinate-1*	*0*.*50* ***±*** *0*.*27*			
*Subordinate-2*	*0*.*18* ***±*** *0*.*22*			
Final status^a^		1.38	3, 28.8	0.27
*Dominant*	*-0*.*16* ***±*** *0*.*27*			
*Subordinate-1*	*-0*.*04* ***±*** *0*.*20*			
*Subordinate-2*	*0*.*11* ***±*** *0*.*22*			
Initial status x Final status^b^		0.99	9, 34.4	0.47
*Dominant* x dominant	*0*.*24* ***±*** *0*.*35*			
*Dominant x subordinate-1*	*0*.*53* ***±*** *0*.*35*			
*Dominant x subordinate-2*	*0*.*28* ***±*** *0*.*37*			
*Subordinate-1 x dominant*	*0*.*96* ***±*** *0*.*43*			
*Subordinate-1 x subordinate-1*	*0*.*56* ***±*** *0*.*34*			
*Subordinate-1 x subordinate-2*	*0*.*37* ***±*** *0*.*36*			
*Subordinate-2 x dominant*	*0*.*07* ***±*** *0*.*37*			
*Subordinate-2 x subordinate-1*	*0*.*18* ***±*** *0*.*31*			
*Subordinate-2 x subordinate-2*	*-0*.*07* ***±*** *0*.*32*			
*b) SOD activity in sperm*				
**Random effects**	***Estimates ± SE***		**Z**	**P**
Aviary	*0*		0	1
Sampling batch	*0*.*14* ***±*** *0*.*16*		0.84	0.20
**Fixed effects**		**F**	**df**	**P**
*Intercept*	*6*.*87* ***±*** *3*.*62*			
Body mass	*-0*.*02* ***±*** *0*.*08*	0.07	1, 39.2	0.79
Tarsus length	*-0*.*01* ***±*** *0*.*16*	0.01	1, 39.2	0.93
Initial status^a^		0.43	3, 39	0.73
*Dominant*	*-0*.*64* ***±*** *0*.*49*			
*Subordinate-1*	*0*.*57* ***±*** *0*.*56*			
*Subordinate-2*	*0*.*02* ***±*** *0*.*44*			
Final status^a^		0.74	3, 39	0.54
*Dominant*	*1*.*01* ***±*** *0*.*59*			
*Subordinate-1*	*0*.*15* ***±*** *0*.*43*			
*Subordinate-2*	*-0*.*02* ***±*** *0*.*48*			
**Initial status x Final status**^b^		**2.29**	**9, 39.3**	**0.036**
*Dominant* x dominant	*-0*.*22* ***±*** *0*.*74*			
*Dominant x subordinate-1*	*0*.*85* ***±*** *0*.*76*			
*Dominant x subordinate-2*	*1*.*11* ***±*** *0*.*78*			
*Subordinate-1 x dominant*	*-2*.*08* ***±*** *0*.*93*			
*Subordinate-1 x subordinate-1*	*-0*.*87* ***±*** *0*.*72*			
*Subordinate-1 x subordinate-2*	*-0*.*09* ***±*** *0*.*76*			
*Subordinate-2 x dominant*	*-1*.*96* ***±*** *0*.*77*			
*Subordinate-2 x subordinate-1*	*0*.*28* ***±*** *0*.*63*			
*Subordinate-2 x subordinate-2*	*0*.*18* ***±*** *0*.*70*			
*c) Proportion of SOD activity in sperm*				
**Random effects**	***Estimates ± SE***		**Z**	**P**
Aviary	*0*		0	1
Sampling batch	*0*.*13* ***±*** *0*.*16*		0.80	0.21
**Fixed effects**		**F**	**df**	**P**
*Intercept*	*3*.*06* ***±*** *3*.*91*			
Body mass	*-0*.*06* ***±*** *0*.*09*	0.43	1, 39.2	0.51
Tarsus length	*-0*.*17* ***±*** *0*.*17*	1.02	1, 39.2	0.32
Initial status^a^		1.20	3, 39	0.32
*Dominant*	*-0*.*46* ***±*** *0*.*53*			
*Subordinate-1*	*0*.*97* ***±*** *0*.*61*			
*Subordinate-2*	*-0*.*20* ***±*** *0*.*48*			
Final status^a^		0.22	3, 39	0.88
*Dominant*	*1*.*20* ***±*** *0*.*64*			
*Subordinate-1*	*0*.*18* ***±*** *0*.*46*			
*Subordinate-2*	*-0*.*11* ***±*** *0*.*52*			
**Initial status x Final status**^b^		**2.40**	**9, 39.4**	**0.028**
*Dominant* x dominant	*-0*.*07* ***±*** *0*.*80*			
*Dominant x subordinate-1*	*0*.*47* ***±*** *0*.*82*			
*Dominant x subordinate-2*	*0*.*81* ***±*** *0*.*84*			
*Subordinate-1 x dominant*	*-2*.*99* ***±*** *1*.*00*			
*Subordinate-1 x subordinate-1*	*-1*.*41* ***±*** *0*.*78*			
*Subordinate-1 x subordinate-2*	*-0*.*42* ***±*** *0*.*82*			
*Subordinate-2 x dominant*	*-2*.*07* ***±*** *0*.*84*			
*Subordinate-2 x subordinate-1*	*0*.*12* ***±*** *0*.*68*			
*Subordinate-2 x subordinate-2*	*0*.*22* ***±*** *0*.*76*			

^a^Relative to subordinate-3 males.

^b^Relative to subordinate-3 x subordinate-3 males. Values in bold indicate significance at α = 0.05; tests of random effects are based on Wald-Z.

## Discussion

In the present study, based on sperm competition models [14, 15], we predicted a negative correlation between social dominance and ejaculate quality, while rank-related differences in ejaculate quality would result from differential antioxidant investment into the protection of the ejaculate (the oxidation-based soma vs. germline allocation trade-off hypothesis). In accordance with our predictions, we found rank-related differences in both the proportion of motile sperm (Fig 1) and ejaculate longevity (Fig 2A). The proportion of motile sperm (e.g. [60, 61, 62]) and ejaculate longevity (e.g. [52]) have been shown to be important determinants of male fertility in various species, and hence ejaculates from subordinate males might have an advantage under sperm competition. Further, we found that dominant males produced more oxidized ejaculates compared to subordinate-1 and -2 males (Fig 1). Our results match the prediction of previous theoretical models [3, 7, 15], although not completely. More specifically, subordinate-1 and -2 males produced the most viable sperm compared to dominant males (Fig 1), while subordinate-3 males produced ejaculates with sperm viability similar to ejaculates of dominant males. Yet, subordinate-3 males produced longer lived ejaculates than males occupying higher social ranks (Fig 2A). The fact that subordinate-3 males produced longer-living ejaculates while having more oxidised and less viable sperm may seem paradoxical. However, in our view, a lower initial sperm viability due to higher sperm oxidation is not incompatible with a greater longevity because sperm quality depends on many factors in addition to sperm redox state. For instance, Ribou and colleagues [25] have shown that ROS production is down-regulated after ejaculation and during storage, thus preventing further oxidation risks. Seminal components can also explain variation in sperm quality across males [63], and seminal fluids have been shown to vary according to mating tactics [64, 65]. It may thus be that other ejaculate components or the down-regulation of ROS production within the sperm cells have compensated for the detrimental effects of an initial higher sperm oxidation, and allowed ejaculates of subordinate-3 males to live longer.

In several species, females store sperm before ovulation [66], and sperm have to survive for hours or even days after insemination to be able to fertilize the ova. For example, Pizzari and colleagues [52] experimentally showed in domestic fowls that spermatozoa stored in the female tract become progressively less numerous with time. Further, when females are inseminated with sperm from two different males, the sperm inseminated in larger numbers have a greater chance to fertilize the eggs than higher-quality sperm when ovulation occurs close to insemination. Yet, once stored in the female storage tubules, higher quality sperm prevail in the long run, and are able to fertilize more eggs than low-quality sperm [52]. Thus, longer-living sperm might increase the likelihood that sperm of subordinate-3 males would be present around the oocyte when the female ovulates, and longer-living sperm could confer a large fertilizing advantage to subordinate-3 males.

We predicted that subordinate males should invest more into defending their ejaculate against OS, and thus should produce higher quality ejaculates. In agreement with our prediction, we found that the proportion of oxidized over total amount of glutathione (GSSG/GSHt), i.e. an indication of the risk of oxidative stress experienced by the cells, was negatively related to the proportion of motile sperm (Fig 4A). More importantly, we found that this ratio varied across social ranks in a way that matched the variation in ejaculate quality (Fig 1). Further, ejaculates from subordinate-3 males were as oxidized as those produced by dominant males (Fig 1), which again supports the hypothesis that subordinate-3 males might be constrained by resource access.

Various non-exclusive explanations can be proposed as to why subordinate-3 males produce more oxidatively stressed ejaculates with a lower proportion of motile sperm. Theory predicts that the pay-offs of large ejaculate expenditure decrease when males face a large number of competitors [14, 67], thus males at the bottom of the hierarchy would be expected to have a lower ejaculate expenditure. However, those models predict that, although they should reduce ejaculate expenditure at each copulation, males facing high intensities of sperm competition should still increasingly invest resources into ejaculate quality [14]. Additionally, while, those models explore how males should strategically expend their sperm according to the number of competitors during a given copulation event, in our study sperm samples were collected via an artificial stimulation (cloacal massage) instead of male-controlled ejaculation in response to an immediate social context. Hence, our results more likely reflect a longer-term investment (over at least one spermatogenesis cycle) into sperm production. Alternatively, the glucocorticoid stress response has been argued to cause oxidative insults [68, 69]. Social status has been shown to be linked to stress, and higher glucocorticoid levels are expressed when either dominant or subordinate individuals exhibit larger allostatic loads [35]. Thus, males having larger allostatic burdens might be unable to invest antioxidant resources in the protection of their ejaculate. In house sparrows, there is no clear link between dominance and glucocorticoid levels, and positive, negative or no associations between social dominance and stress have been found [70–72]. Finally, social status-related differences in the access to resources could result in differential access to key antioxidant resources [73, 74]. Thus, subordinate males might have restricted resource access that constrains their investment into the protection of their ejaculates without largely compromising their own body condition.

After changing the social environment for all males, we found that males adjusted their sperm quality to match their new social status (Fig 3). Indeed, we found that males that were initially dominant, subordinate-1 or subordinate-2 adjusted the proportion of motile sperm according to our predictions (Fig 3A–3C). Flexibility in sperm traits given rapid changes in social environments has been reported in previous studies [9, 10, 75], although the physiological proximate mechanism underlying such plasticity remained to be identified. Here, we found that the proportion of motile sperm positively correlated not only with the absolute amount of the endogenous enzymatic antioxidant superoxide dismutase (SOD) activity in the ejaculate, but also with the proportion of SOD activity in sperm relative to the SOD activity in blood (Fig 4B). Further, adjustments in ejaculate quality were paralleled by adjustments in antioxidant investment in the ejaculate (Fig 3). Such results support our hypothesis that antioxidants are a key resource to be strategically allocated in the soma vs. the germline, and hence they are a likely proximate physiological mechanism mediating social status-related difference in ejaculate quality.

Interestingly, we found no apparent somatic cost, as measured by lipid peroxidation, before or after experimentally manipulating the social status (see results). In house sparrows, dominant males have been previously shown to have higher mate guarding rates [36, 76], higher access to females [36, 76], and to exhibit larger badges [77], thus showing a greater investment into pre-copulatory/somatic functions. Further, oxidative stress is also likely to affect other molecules than membrane lipids, thus causing disrupted physiological pathways or higher ageing rates [17]. As a consequence, such somatic costs could be observed in other tissues than blood (e.g. [78]), and further experiments should explore how somatic costs of reproduction are paid in different body compartments. Alternatively, our results might reflect strong physiological constrains on somatic maintenance, while males may be able to afford more flexibility in the extent to which they protect their ejaculates against oxidation. Finally, subordinate-3 males produced ejaculates with a higher proportion of motile sperm and invested a greater proportion of SOD activity in their ejaculates as they moved up in the hierarchy (Fig 3D), supporting the idea that access to resources constrains subordinate-3 males in the development of their reproductive tactic. As they acquire a higher social status, such constraints may be lifted, and they increase their investment into ejaculate quality.

Promiscuity is common across animal taxa, and studies exploring the mechanisms modulating male fertility are essential to understand how sexual selection acts upon male reproductive strategies. There are a variety of factors causing oxidative stress, e.g. immune response, metabolic processes, pollutants [17]. As a consequence, oxidative stress is an unavoidable physiological cost [33], and may thus be a major cost of reproduction [18, 19] as well as a universal constraint to life history evolution[55, 79]. To our knowledge, we show, for the first time in a socially monogamous bird species, that males develop their reproductive tactics along a linear social hierarchy, and remarkably that oxidative stress is one likely proximate physiological mechanism underlying the development of these tactics. Oxidative stress is known to have deleterious effects on sperm of various taxa [23, 26, 32], and thus we suggest that in species where males face sperm competition strategic antioxidant allocation could be an important physiological mechanism modulating ejaculate quality. Further, it remains to be explored how the glucocorticoid stress response and/or differences in resource access interact with OS, thus affecting patterns of antioxidant allocation into pre- and post-copulatory traits. Finally, we suggest that future theoretical models should include a minimal cost of bodily functions maintenance that guarantees male condition and survival (e.g. allostasis, resource access), which could potentially limit resource allocation to the germline.

## Supporting information

S1 Data Supporting data. (XLSX)

S1 File Supplementary material and methods. (DOCX)
