# Supplementary material for: Antioxidant allocation modulates sperm quality across changing social environments
Source: PLoS One. 2017 May 4;12(5):e0176385. doi: 10.1371/journal.pone.0176385 (PMC5417513; doi:10.1371/journal.pone.0176385)
Supplement: S1 File — (DOCX) [file pone.0176385.s002.docx]

Supporting Information

Antioxidant allocation modulates ejaculate quality across changing social environments

Alfonso Rojas Mora^1^, Magali Meniri^1^, Ophélie Gning^1^, Gaëtan Glauser^2^, Armelle Vallat^2^ & Fabrice Helfenstein^1*^

^1^Laboratory of Evolutionary Ecophysiology, Institute of Biology, Faculty of Sciences, University of Neuchatel, Neuchatel, Switzerland

^2^Neuchatel Platform of Analytical Chemistry, Institute of Chemistry, Faculty of Sciences, University of Neuchatel, Neuchatel, Switzerland

^*^Correspondence to: FH. Rue Emile-Argand 11, Institute of Biology, Faculty of Sciences, University of Neuchatel, Neuchatel, Switzerland. E-mail: fabrice.helfenstein@free.fr

**Supplementary materials and methods**

Individuals

We trapped a total of 60 male and 60 female house sparrows using mist-nets in western Switzerland (Neuchatel, Biel, Bern, and Fribourg), between the 8th and the 17th of April 2014. After trapping, we measured body mass, tarsus length, and wing length prior to transferring them into 15 mixed-sex outdoor aviaries at the Ethological Station, University of Bern (Switzerland). At the aviaries, each individual received on each leg a colour ring unique to its aviary (Males: red, white, orange, yellow; Females: blue, green, violet, pink;). A total of 4 males and 4 females were held in each aviary for ca. 2 months. During this period, they received a controlled amount of food (300 gr per aviary every second day; germinated barley seeds 25 %, egg protein supplement (Quicko® Classic, Germany) 15%, and a mix of seeds for canaries 60%) and each aviary contained an artificial pond with running water.

Experimental design and samples collection

After four weeks of acclimatization, we transferred the females into a separate aviary during 4 days. On the day the females were removed, we took a blood and a sperm sample from each male, followed by a second sperm sample the day after and a third sperm sample after 4 days of being sexually rested. Then, males were shuffled between aviaries according to their current status, in a way that optimized the expected number of males moving up and down the hierarchy (Table. S1). Once the males were shuffled across aviaries, females were re-introduced in their initial aviaries, and we let the new groups in the aviaries for another 18 days. After this 18-day period allowing males to establish their new hierarchical ranks, females were released into their localities of origin. On the day females were released, blood and sperm samples were taken from males, and further sperm samples were taken on the second and the fourth day after female release. We used the data on the fourth day of sperm sampling, to control for any possible fresh sperm effect due to differential access to females by males. For logistic reasons, we divided the 60 males into three batches consisting of 20 males (5 aviaries), which were processed with a difference of 5 days between each batch. At the end of the experiment, all birds were released into their localities of origin. The experiments were conducted following the animal welfare legislation under the permit BE41/12 for animal research and the permit WTH/g-525/14 for animal detention, both granted by the office of agriculture and nature of canton Bern, Switzerland.

Behavioural observations

To establish the hierarchy of the males in each aviary, we recorded a total of 13 hours of observations before the manipulation and 10 hours after the manipulation in each aviary. Males were observed interacting at the feeder, which consisted in a seed dispenser with two feeding stations, mounted on top of a plastic plate covered with a plastic mesh. Any spilt seeds were inaccessible and birds had to compete for the two feeding sites at the feeder. Before recording the videos, we removed the feeders for 1.5 hours to increase males’ motivation over food. We then located a GoPro camera at ca. 60 cm from the feeder to record all the male-male interactions for one hour as soon as the feeders were put back into the aviaries. Using the dyads at each aviary (acclimation: 82 dyads per aviary on average, range 31-235; post-shuffling: 100 dyads per aviary on average, range 39-233), we computed each male's David's score as a proxy for their social rank within each group [3].

Blood samples

We obtained a blood sample on the first and last day of experiment by punctuating the alar vein with a 27-gauge needle, and approximately 150 µL of blood were collected in a heparinized capillary (Microvette CB300, Sarsted AG & Co, Germany). Blood samples were centrifuged for 5 min at 4000 RPM and 4°C, and then plasma and erythrocytes pellets were removed and stored at -80°C.

Sperm samples

We obtained ejaculates (ca. 2.5 µL) by gently massaging male’s cloaca [7]. Right after collecting the ejaculates, 0.2 µL of sperm were dissolved into 40 µL of pre-warmed DMEM (Dulbecco modified eagle medium; Sigma Aldrich, USA), and then transferred to a 20 µm depth chamber slide (Leja Products B.V., The Netherlands). We video recorded sperm for 75 seconds at 13 frames per second using a Toshiba CMOS HD camera (TOSHIBA Corporation, Japan) on an Olympus BX43 microscope (Olympus Co., Japan) at 100x magnification under a phase contrast 3, while temperature was maintained at 40°C using a heating plate on the microscope (Minitube HT200 W, MINITÜB GmbH, Germany). The time between the ejaculate collection and the start of the video was recorded. We also collected a subsample of 1 µl of sperm into 9 µL of PBS for MDA assessment, and we stored it at -80°C.

We estimated curvilinear velocity (VCL), average path velocity (VAP), straight-line velocity (VSL), and proportion of swimming sperm from 4 video segments of 2.5 seconds at 0 (hereafter referred to as "initial swimming speed" and "initial proportion of motile sperm"), 15, 30, 45, 60 and 90 seconds after recording started, using a Computer Assisted Sperm Analyser plug-in [6] for ImageJ [5]. Due to technical problems, the first 2 seconds of video were discarded. A preliminary analysis showed that 2.5 seconds segments maximized the number of sperm tracks detected, while avoiding inflating the percentage of motile sperm due to sperm cells entering the recording field. The minimum and maximum size for a sperm cell were set to 70 and 100 pixels respectively. The minimum tract time was set to 1.31 seconds, and the search radius to 35 pixels. Such settings minimized the risk of counting sperm cells that would enter into the filming area for a short period, while avoiding merging trajectories of crossing sperm into a single track. Sperm cells having a VSL<5 µm/s, a VCL<15 µm/s, or a VAP<10 µm/s were counted as immotile. From the measurements, we obtained the initial sperm swimming speed, sperm swimming endurance (rate at which initial swimming speed decreases through time), and mortality (rate at which initial proportion of swimming sperm decreases through time). Sperm endurance and sperm mortality were derived as individual slopes from a mixed model including curvilinear velocity VCL or the proportion of motile sperm, respectively, as dependent variables, and time (0, 15, 30, 45, 60 and 90 seconds) as a continuous independent variable, with individual identity in interaction with time as a random factor.

Oxidative stress and antioxidant defences

*Lipid peroxidation*

We assessed the amount of lipid peroxidation by determining the circulating levels of malondialdehyde (MDA) in plasma, erythrocytes, and sperm. Concentrations of MDA, formed by the β-scission of peroxidised fatty acids, were assessed using UHPLC with fluorescence detection, following Moselhy et al. [4] and Agarwal et al. [1] with modifications. All chemicals were of analytical or HPLC grade, and chemical solutions were prepared using ultra-pure water (Milli-Q Synthesis; Millipore Corporation, Billerica, MA, USA). To a (plasma or RBC / sperm) 5/8 μl aliquot of sample (0.8 µL of sperm in 7.2 µL of PBS; 0.5 µL of erythrocytes in 4.5 µL of PBS; 5µL of plasma) or standard (1,1,3,3-tetraethoxypropane, TEP; Sigma Aldrich, USA), 155/152 μl of water, 40 μl of trichloroacetic acid 5% (TCA), and 20 μl thiobarbituric acid (TBA; Sigma Aldrich, USA) solution (42 mM) were added in a 1.5 mL tube. Samples were then vortex mixed for 5 seconds and centrifuged for 14 minutes at 14000 rpm and 4°C. 205 µL of the epiphase was transferred to 2 ml capacity conical-bottom screw-top microcentrifuge tubes, vortex mixed for 5 seconds, then heated at 100°C for exactly 1 hour in a dry bath incubator to allow formation of MDA-(TBA)_2_ adducts. Samples were then cooled on ice for 5 minutes, and 150 µL of butanol (Sigma Aldrich, USA) were added. Then, the tubes were vortex mixed for 10 seconds and centrifuged for 10 minutes at 14000 rpm and 4°C. Then, 120 µL of the epiphase was recovered in 1.5 mL tubes. A second extraction of the derivatized solution was done by adding 150 µL of butanol, vortex mixed for 10 seconds and centrifuged for 10 minutes at 14000 rpm and 4°C. Then, 140 µL of the epiphase was pooled with the previously recovered epiphase, and then evaporated in a SpeedVac for 60 minutes at 35ºC. Finally, the product was re-suspended in 90 µL of methanol 30% (Sigma Aldrich, USA), and the tubes were sonicated for 5 seconds and then vortex mixed for 10 seconds. A 70 μl aliquot was collected and transferred to an HPLC vial for analysis. Samples (5 μl) were injected into a Dionex Ultimate 3000 Rapid Separation LC system (Dionex Corporation, California, USA) fitted with a Waters (Milford Massachusetts, USA) Acquity UPLC® BEH C18 column (1.7μm, 2.1 x 50 mm) maintained at 30°C. Separation was achieved using gradient elution at a flow rate of 0.4 ml/min with solvent A being 0.05% acetic acid buffered at pH 6 with ammonium hydroxide and solvent B acetonitrile. The gradient was as follows: linear increase from 5% to 100% solvent B over 5 min, followed by 100% solvent B for 1.5 min and re-equilibration at initial conditions (5% B) for 3.2 min. The total analysis time was 9.7 min. The auto-sampler syringe was washed with 700 μl of solvent B after each injection. Data were acquired using a fluorescence detector set at 515 nm (excitation) and 553 nm (emission). For calibration, a standard curve was prepared using a TEP stock solution (5 μM in 40% ethanol) serially diluted using pure water. All the samples were processed blindly of both the identity of the individual and social rank.

*Superoxide dismutase activity*

We also determined superoxide dismutase activity perm ml of tissue in sperm and blood Cayman Chemical, (USA) commercial kit with minor modifications. Specifically, with used dilution of 1:400 for erythrocytes and 1:160 for sperm. Moreover, all the samples were done in duplicate and the %CV was maintained bellow 15% (average %CV: 10.7% for sperm and 9.9% for erythrocytes). All the samples were processed blindly of both the identity of the individual and social rank.

*Glutathione concentrations*

Finally, we determined the levels of glutathione, an intracellular antioxidant in both its reduced (GSH) and oxidized (GSSG) forms, both in sperm and red blood cells following a modified protocol based on Bouligand et al. [2]. We{Bouligand, 2006 #196} mixed (sperm/RBC) 3/10 µL of aliquot (0.3µL sperm in 2.7 µL of PBS; 1µL of erythrocytes in 9 µL of PBS), 5/25 µL of TCA 5%, 5µL of glutathione ethyl ester (GSHee; Sigma Aldrich, USA) as internal standard (2.25 µg/mL or 20 µg/mL respectively), and 62/0 µL of pure water in a 1.5 mL tube. Then, the solutions were vortex mixed, kept ice-cold for 5 minutes, and centrifuged for 14 minutes at 140000 rpm and 4ºC. After centrifugation, 50/20 µL of the epiphase were diluted in 100/980 µL of water and then 100 µL of the dilution was transferred into glass HPLC vials. Samples (5 μl) were injected on a Dionex Ultimate 3000 Rapid Separation LC system (Dionex Corporation, California, USA) coupled to a 4000 QTRAP mass spectrometer (Sciex, Toronto, Canada) equipped with a Turbo V source. A Waters Acquity UPLC® BEH HSS T3 column (1.8μm, 2.1 x 100 mm) was employed at a flow rate of 0.4 ml/min using the following solvent system: solvent A = milli-Q H_2_O with 0.05% formic acid, solvent B = acetonitrile with 0.05% formic acid. The gradient was as follows: 0-20% B in 2 min, 20-100% B in 3 min, 100% B for 3 min, back to 0% B for 5 min. Total analysis time was 13 min. Mass spectrometry detection was achieved using multiple reaction monitoring (MRM) transitions in positive ionization mode (Table S2). A dwell time of 50 ms was applied for all transitions. Ion source parameters were as follows: capillary voltage 5.5 kV, nebulizing gas (GS1) 45 psi, drying gas (GS2) 25 psi at 550°C, curtain gas (CUR) 15 psi. For quantification, a standard curve containing GSH (Sigma Aldrich, USA) and GSSG (Sigma Aldrich, USA) at 0.002, 0.02, 0.1, 0.5, and 2 μg/mL and GSHee at a constant concentration of 0.05 µg/mL was made. All the samples were processed blindly of both the identity of the individual and social rank.

**References**

1. Agarwal, R. & Chase, S.D. (2002). Rapid, fluorimetric–liquid chromatographic determination of malondialdehyde in biological samples. *Journal of Chromatography B*, 775, 121-126.

2. Bouligand, J., Deroussent, A., Paci, A., Morizet, J. & Vassal, G. (2006). Liquid chromatography–tandem mass spectrometry assay of reduced and oxidized glutathione and main precursors in mice liver. *Journal of Chromatography B*, 832, 67-74.

3. Gammell, M.P., de Vries, H., Jennings, D.J., Carlin, C.o.M. & Hayden, T.J. (2003). David's score: a more appropriate dominance ranking method than Clutton-Brock et al.'s index. *Animal Behaviour*, 66, 601-605.

4. Moselhy, H.F., Reid, R.G., Yousef, S. & Boyle, S.P. (2013). A specific, accurate, and sensitive measure of total plasma malondialdehyde by HPLC. *Journal of Lipid Research*, 54, 852-858.

5. Schneider, C.A., Rasband, W.S. & Eliceiri, K.W. (2012). NIH image to ImageJ: 25 years of image analysis. *Nature Methods*, 9, 671-675.

6. Wilson-Leedy, J.G. & Ingermann, R.L. (2007). Development of a novel CASA system based on open source software for characterization of zebrafish sperm motility parameters. *Theriogenology*, 67, 661-672.

7. Wolfson, A. (1952). The Cloacal Protuberance: A Means for Determining Breeding Condition in Live Male Passerines. *Bird-Banding*, 23, 159-165.

Table S1.

Shuffling scheme of males across aviaries to experimentally manipulate their social status.

Table S2.

Analyte-dependent mass spectrometry parameters. DP, declustering potential, CE, collision energy, CXP, collision cell exit potential.

| **Analyte** | **Transitions** | **DP (V)** | **CE (eV)** | **CXP (V)** |
| --- | --- | --- | --- | --- |
| GSH | 308/162 | 51 | 25 | 14 |
|  | 308/179 | 51 | 27 | 14 |
|  | 308/84 | 51 | 45 | 10 |
| GSSG | 307/130 | 46 | 29 | 10 |
|  | 307/84 | 46 | 35 | 10 |
| GSEE | 336/207 | 56 | 19 | 18 |
|  | 336/190 | 56 | 23 | 16 |
